# Supplementary material for: Exercise Intensity in Patients with Cardiovascular Diseases: Systematic Review with Meta-Analysis
Source: Int J Environ Res Public Health. 2021 Mar 30;18(7):3574. doi: 10.3390/ijerph18073574 (PMC8037098; doi:10.3390/ijerph18073574)
Supplement: Supplementary file 1 [file ijerph-18-03574-s001.pdf]

Supplementary Material

Contents

1. MEDLINE Search Strategy..2
2. Summary of Study Characteristics...3
3. Outcome of the Risk of Bias Assessment...5
4. List of references for included studies 7

Figure S1. *Search Strategy for MEDLINE Database, searched from inception till January 2021.*

Key concepts	**Concept 1-** Patient, Population<br>**Patients with cardiac diseases (OR)**		**Concept 2 -** Intervention (or Exposure)<br>**Exercise in Cardiac Rehabilitation (OR)**		**Concept 3 -** Comparison (or Control) if appropriate<br>**Exercise intensity/Programs (OR)**		**Concept 4 -** Outcome<br>**Aerobic capacity (OR)**
**Free text terms / natural language terms**	Myocard* near/5 isch*mi*<br>Isch*mi* near/5 heart<br>Myocard* near/5 infarct*<br>Heart near/5 infarct*<br>Angina<br>Coronary near/5 disease* or bypass or thrombo* or angioplast*<br>(Percutaneous next coronary near/2 interven* or revascular*<br>Angioplast*<br>coronary or arterial near/4 dilat*<br>Endoluminal next repair*<br>Stent*<br>Pci or ptca<br>Atherectom*<br>Acute next coronary next syndrom*	**AND**	Rehabilitat*<br>Physical* near/5 fit* or train* or therap* or activit*<br>train* near/5 strength* or aerobic* or exercise*<br>exercise* or fitness near/3 treatment or intervent* or program*<br>Kinesiotherap*<br>patient* near/5 educat*<br>lifestyle or life-style near/5 interven* or program* or treatment*<br>self near/5 manag* or care or motivate*<br>Psychotherap*<br>Psycholog* near/5 intervent*<br>counselling or counseling<br>behavior* or behaviour* near/5 modify or modificat* or therap* or change<br>psycho-educat* or psychoeducat*<br>motivat* near/5 (intervention or interv*<br>health near/5 educat*<br>psychosocial or psycho-social<br>cognitive near/2 behav*	**AND**	Exercise Test/methods*<br>High-Intensity Interval Training*<br>HIIT<br>HIIT or HIT<br>Exercise intensit*<br>Exercise next intensit*<br>Exercise near/5 intensit*<br>Exercise Intensity Program*<br>intensit* near/5 interven* or program* or treatment*<br>intensity* near/5 interven* or program* or treatment*<br>intensit* near/5 method*<br>intensit* near/5 exercise*<br>intensit* near/5 program*<br>Moderate continuous training<br>Moderate-intensity continuous training<br>moderate near/4 training<br>MCT<br>moderate continuous training near/5 methods*<br>moderate continuous training near/5 standards*<br>*rehabilitation near/5 exercise*<br>*rehabilitation near/5 program*<br>Home-based<br>Home-based near/5 methods*<br>Home-based near/5 satandards*<br>Aerobic Exercises<br>Anaerobic Exercises<br>No exercise	**AND**	Heart Failure next mortality<br>Heart Failure near/5 mortality<br>Cardiovascular Capacity*<br>Oxygen Consumption/physiology*
**Controlled vocabulary terms / Subject terms**	Intervention (MeSH)<br>Coronary Artery Bypass (MeSH)<br>Percutaneous Coronary Intervention (MeSH)<br>Angioplasty (MeSH)<br>Stents (MeSH)<br>Atherectomy (MeSH)		Exercise Therapy (MeSH)<br>Sports (MeSH)<br>Physical Exertion (MeSH)<br>Exercise (MeSH)<br>Rehabilitation (MeSH)<br>Physical Education and Training (MeSH)<br>Patient Education as Topic (MeSH)<br>Self Care (MeSH)<br>Psychotherapy (MeSH)<br>Counseling (MESH)<br>Health Education (MeSH)		Exercise Tolerance (MeSH)<br>High Intensity Interval Training/methods (MeSH)<br>High Intensity Interval Training/standards (MeSH)<br>Telerehabilitation (MeSH)<br>Telerehabilitation/methods (MeSH)<br>Telerehabilitation/standards (MeSH)<br>Cardiac Rehabilitation/methods (MeSH)<br>Cardiac Rehabilitation/standards (MeSH)<br>High Intensity Interval Training/standards (MeSH)		Surveys and Questionnaires (MeSH)<br>Cardiovascular Diseases/epidemiology (MeSH)<br>Cardiovascular Diseases/mortality (MeSH)<br>Morbidity/trends (MeSH)<br>Motor Activity/physiology*(MeSH)<br>Prevalence (MeSH)<br>Risk Factors (MeSH)<br>Sedentary Lifestyle (MeSH)<br>Oxygen Consumption/physiology (MeSH)<br>Predictive Value of Tests (MeSH)<br>Reproducibility of Results (MeSH)<br>Recovery of Function (MeSH)

__Table S2__. Summary of Study Characteristics.

		Participant characteristics			Rehabilitation protocols										Primary Outcome	
**Study**	**Year**	**n (men)**	**Age**	**Conditions**	**Length**	**Freq.**	**Exercise duration**	**Intensity**	**Mode**	**Type**	**Sup.**	**Prog.**	**Res.**	**Comp.**	**ΔVO2R**	**ΔVO2AT**
Abolahrari-Shirazi et al.	2018	25 (NS)	56.76 (8.71)	PCI	7	3	W:5; A:30; C:5	40-70%VO2p	CY+AE+TM+RES	Cont	CL/H	Y	Y	Y	8.2	
		25 (NS)	57.64 (7.85)	PCI	7	3	W:5; A:45; C:5	40-70%VO2p	CY+AE+TM	Cont	CL/H	Y	N	Y	9.4	
		25 (NS)	57.32 (9.41)	PCI								N	N	Y	1.8	
Blumenthal et al.	2005	48 (31)	62±11	IHD EMI	16	3	W:10; A:35; C:10	70-85%HRR	WK	Cont	CL	N	N	N	1.9	
		44 (29)	63±12	IHD EMI	16	1			STM		CL	N	N	N	0.3	
		42 (32)	63±9	IHD EMI								N	N	N	-0.3	
Chuang et al.	2005	17 (15)	64±8	CABG	12	2	A:30	70-80%HRp, 60-70%VO2p, RPE 11-15	TM	Cont	CL	N	N	N	4.76	
		15 (13)	69±12	CABG								N	N	N	1.72	
Ghroubi et al.	2013	16 (NS)	59±6	CABG	8	3	W:10; A:20; C:10	70%HRR	CE	Cont	CL	N	N	N	1.70	
		16 (NS)	59±2	CABG	8	3	W:5; A:20;	70%HRR (20-30% Peak torque)	RES	Cont/Int	CL	N	Y	N	4.00	
Giallauria et al.	2006	22 (16)	55±8	MI	12	3	W:5; A:30; C:5	60-85%VO2p	CE+CY	Cont	CL/H	Y	N	N	4.2	
		22 (17)	54±10	MI	12	3	W:5; A:30; C:5	60%VO2p	CE	Cont	CL	Y	N	N	3.8	
Giallauria et al.	2009	26 (2)	58±8	MI	12	3	W:5; A:30; C:5	60-70%VO2p	CE	Cont	CL	N	N	N	4.3	2.3
		26 (2)	57±10	MI								N	N	N	-2	-1.7
Giallauria et al.	2011	37 (28)	61±7	MI	26	3	W:5; A:30; C:5	60-70%VO2p	CE	Cont	CL	N	N	Y	4	
		26 (23)	52±10	MI	26							N	N	N	1	
Giallauria et al.	2013	25 (22)	54±7	MI	26	3	W:5; A:30; C:5	60-70%VO2p	CE	Cont	CL	N	N	N	4.00	
		21 (18)	54±9	MI								N	N	N	1.00	
Kitzman et al.	2013	32 (NS)	70±7	FMD CAS	16	3	W:10; A:20(WK)+20(E); C:10	40/50-70%HRR	WK, AE, CE	Cont	CL	Y	N	N	1.6	

		31 (NS)	70±7	FMD CAS	16	2						N	N	N	-0.2
Kraal et al.	2013	25 (21)	56±9	PCI CABG	12	2	A:45-60	70-85%HRp	TM, CE	Cont	CL	N	N	N	2.40
		25 (22)	61±8	PCI CABG	12	2	A:45-60	70-85%HRp	TM, CE	Cont	CL/H	N	N	N	3.20
Kubo et al.	2004	24 (21)	59±12	MI	12	3	A:320	60-70%HRp	TM, CE	Cont	CL	N	N	N	2.9
		24 (17)	62±12	MI	12							N	N	N	-0.3
Legramante et al.	2017	43 (NS)	60±9	CABG	2	6x2	A:30	75-85% HRp	WK, CAL, CE	Cont	CL	Y	Y	N	2.6
		39 (NS)	58±8	CABG	2	6x2	A:30	75-85% HRp	WK, CAL	Cont		Y	N	N	0.9
Tamburus et al.	2016	15 (NS)	57±7	CAD	16	3	W:10; A: 30-40; C:10	70-110% VO2VAT	CE	Int	CL	Y	N	N	1.51
		17 (NS)			16							N	N	N	-1.86
		15 (NS)	57±7	None	16	3	W:10; A: 30-40; C:10	70-110% VO2VAT	CE	Int	CL	Y	N	N	1.95
		17 (NS)		None	16							N	N	N	-1.43
Villelabeitia et al.	2017	37 (29)	58±11	CAD	8	3	W:5-12; A:15-30; C:5-13	104.5% ± 22.2% VO2p (1ºmonth) and 134.5% ± 29.7% VO2p (2ºmonth)	CE	Int	CL	Y	N	N	4.5
		36 (33)	58±11	CAD	8	3	W:5-12; A:15-30; C:5-13	64.2% ± 8.5% VO2p (1ºmonth) and 69.5% ± 8.7% VO2p (2ºmonth)	CE	Cont	CL	Y	N	N	2.46
Wu et al.	2006	18 (NS)	63±7	CABG	12	3	W:10; A:30-60; C:10	60-85 %HRp	TM, CE	Cont	CL	N	N	N	8.50
		18 (NS)	61±8	CABG	12	3	W:10; A:30-60; C:10	60-85 %HRp, RPE 11-13	WK	Cont	H	N	N	N	6.50
		18 (NS)	62±10	CABG								N	N	N	3.50
Zheng et al.	2008	27 (NS)	NS	MI	26	3	W:15; A:30; C:15	100% AT	CE	Cont	CL	N	N	N	3.10
		30 (NS)	NS	MI	26							N	N	N	0.3

NS, not stated/missing. n(men) presented as the sample size (number of men). Age presented as mean±SD years. Conditions: MI, myocardial infarction. CABG, coronary artery bypass graft. PCI, percutaneous coronary intervention. CAD, coronary artery disease. IHD, ischemic heart disease. EMI, exercise-induced myocardial ischemia. FMD, endothelial-dependent flow-mediated arterial dilation. CAS: carotid artery stiffness. Rehabilitation protocols: Length presented as no. of weeks. Frequency (Freq.) presented as sessions per week. Exercise Duration: presented as minutes per session: W, warm-up. A: aerobic component (interval programs presented as interval x duration). C, cool-down. SMT: stress management training. R, recovery. wk, week. Intensity: %HRp, % peak heart rate. %HRR, % heart rate reserve. %VO_2peak, % peak oxygen uptake. %AT/VT, % of anaerobic/ventilatory threshold. RPE, rating of perceived exertion. Mode: TM, treadmill. CE, cycle ergometer. AE, arm ergometer. RES, resistance training protocol. WK, walking/jogging. CY, cycling, training. CAL: Calisthenics. Type: Cont, continuous training. Int, interval. Supervision (Sup.), level of

monitoring/supervision: CL, clinic-based. H, home-based. Progressive (Prog.), whether aerobic exercise intensity was re-evaluated during the program: Y, yes. N, no. Resistance exercises (Res.): Y, yes. N, no. Comprehensive rehabilitation (Comp.), exercise training plus education and risk factor management: Y, yes; N, No. Outcomes: ΔVO_2R, change in relative VO_2peak (presented as $mL \cdot kg^{-1} \cdot min^{-1}$).

Figure S3*. Outcomes of the risk of bias assessment.*

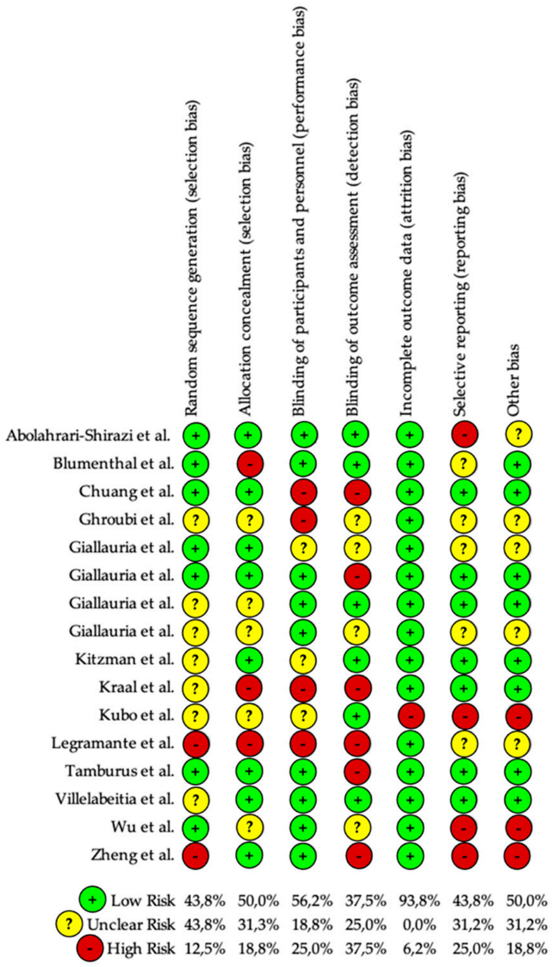

For references, see Supplementary Material S4.

S4. List of references for included studies

1. Abolahrari-Shirazi, S.; Kojuri, J.; Bagheri, Z.; Rojhani-Shirazi, Z. Efficacy of combined endurance-resistance training versus endurance training in patients with heart failure after percutaneous coronary intervention: A randomized controlled trial. Journal of Research in Medical Sciences. 2018;23.
2. Blumenthal, J.A.; Sherwood, A.; Babyak M, et al. Effects of Exercise and Stress Management A Randomized Controlled Trial. American Medical Association 2005;293(13)
3. Chuang, T.; Sung, W.; Lin, C. Application of a virtual reality-enhanced exercise protocol in patients after coronary bypass. Arch Phys Med Rehabil 2005;86(10):1929-32.
4. Ghroubi, S.; Elleuch, W.; Abid L, et al. Effects of a low-intensity dynamic-resistance training protocol using an isokinetic dynamometer on muscular strength and aerobic capacity after coronary artery bypass grafting. Ann Phys Rehabil Med 2013;56(2):85-101. doi: 10.1016/j.rehab.2012.10.006
5. Giallauria, F.; De Lorenzo, A. et al. Long-term effects of cardiac rehabilitation on end-exercise heart rate recovery after myocardial infarction. European Journal of Cardiovascular Prevention & Rehabilitation 2006; 13(4): 544-550
6. Giallauria, F., Lucci, R.; D'Agostino, M. et al. Two-year multicomprehensive secondary prevention program: Favorable effects on cardiovascular functional capacity and coronary risk profile after acute myocardial infarction. J Cardiovasc Med 2009;10(10):772-80. doi: 10.2459/JCM.0b013e32832d55fe
7. Giallauria, F.; Cirillo, P.; D'Agostino, M. et al. Effects of exercise training on high-mobility group box-1 levels after acute myocardial infarction. J Cardiac Failure 2011;17(2):108-14. doi: 10.1016/j.cardfail.2010.09.001
8. Giallauria, F.; Acampa, W.; Ricci, F. et al. Exercise training early after acute myocardial infarction reduces stress-induced hypoperfusion and improves left ventricular function. Eur J Nuc Med Molecul Imag 2013;40(3):315-24. doi: 10.1007/s00259-012-2302-x
9. Kitzman, D.W.; Brubaker, P.H. et al. Effect of endurance exercise training on endothelial function and arterial stiffness in older patients with heart failure and preserved ejection fraction: a randomized, controlled, single-blind trial. Journal of the american college of cardiology 2013; 62(7):584-592
10. Kraal, J.J.; Peek, N.; Van den Akker-Van Marle, M.E. et al. Effects of home-based training with telemonitoring guidance in low to moderate risk patients entering cardiac rehabilitation: short-term results of the FIT@Home study. Eur J Prev Cardiol 2014;21:26-31. doi: 10.1177/2047487314552606
11. Kubo, N.; Ohmura, N.; Nakada, I. et al. Exercise at ventilatory threshold aggravates left ventricular remodeling in patients with extensive anterior acute myocardial infarction. American Heart Journal 2004; 144(1): 113-120
12. Legramante, J.M.; Iellamo, F.; Massaro, M. et al. Effects of residential exercise training on heart rate recovery in coronary artery patients. American Journal Of Physiology. Heart And Circulatory Physiology 2007; 292(1): H510-H515
13. Tamburús, N.Y.; Kunz, V.C.; Salviati, M.R. et al. Interval training based on ventilatory anaerobic threshold improves aerobic functional capacity and metabolic profile: a

randomized controlled trial in coronary artery disease patients. European Journal Of Physical And Rehabilitation Medicine 2016; 52(1):1-11

14. Villelabeitia-Jaureguizar, K.; Vicente-Campos, D.; Senen, A.B. et al. Effects of high-intensity interval versus continuous exercise training on post-exercise heart rate recovery in coronary heart-disease patients. International Journal of Cardiology 2017; 244: 17-23
15. Wu, Y.T.; Wu, Y.W.; Hwang, C.L. et al. Changes in diastolic function after exercise training in patients with and without diabetes mellitus after coronary artery bypass surgery. A randomized controlled trial. Eur J Phys Rehabil Med 2012;48(3):351-60.
16. Zheng, H.; Luo, M.; Shen, Y. et al. Effects of 6 months exercise training on ventricular remodelling and autonomic tone in patients with acute myocardial infarction and percutaneous coronary intervention. J Rehabil Med 2008;40(9):776-9. doi: 10.2340/16501977-0254
